# Detection of *Salmonella* from animal sources in South Africa between 2007 and 2014

**DOI:** 10.4102/jsava.v89i0.1643

**Published:** 2018-11-07

**Authors:** Awoke K. Gelaw, Palesa Nthaba, Itumeleng Matle

**Affiliations:** 1 Agricultural Research Council, Irene, South Africa

## Abstract

Retrospective laboratory-based surveillance was conducted on *Salmonella* serotypes isolated from various animal species from 2007 to 2014 at the Agricultural Research Council, Onderstepoort Veterinary Research Institute, South Africa. During the surveillance period, 1229 salmonellae isolations were recorded. Around 108 different serotypes were recovered from nine different food and non-food animal host species. The three most common serotypes were *Salmonella enterica* subspecies *enterica* serotype Heidelberg (*n* = 200), *Salmonella enterica* subspecies *enterica* serotype Enteritidis (*n* = 170) and *Salmonella enterica* subspecies *enterica* serotype Typhimurium (*n* = 146). These were followed by *Salmonella enterica* subspecies *enterica* serotype Anatum (*n* = 62) and *Salmonella enterica* subspecies *enterica* serotype Infantis (*n* = 57). *Salmonella enterica* subspecies *enterica* serotype Schwarzengrund and *Salmonella enterica* subspecies *enterica* serotype Muenchen were recovered in 50 and 48 cases, respectively. Of the total number of isolations recorded during the period under review, 871 (70.8%) occurred in poultry and other birds, 162 (13.2%) in horses, 116 (9.4%) in cattle, 26 (2.1%) in sheep and goats, 22 (1.8%) in rhinoceroses, 16 (1.3%) in pigs, 8 (0.6%) in crocodiles, 6 (0.5%) in cats and 6 (0.5%) in leopards. Food animals accounted for 83.5% of the total isolations, with cattle and poultry representing approximately 72.7%. Forty-two (3.4 %) isolates were found from non-food animals that include rhinoceroses (*n* = 22), crocodiles (*n* = 8), leopards (*n* = 6) and cats (*n* = 6). *Salmonella* Heidelberg was the most frequently isolated serotype, whereas *S.* Typhimurium had the widest zoological distribution. Clinical laboratory isolation of different *Salmonella* serotypes from various hosts may aid in recognising the threat to livestock, public and environmental health. Moreover, it may also highlight the potential zoonotic and food safety risk implications of the detected *Salmonella* serotypes.

## Introduction

*Salmonella* infection (salmonellosis) is a common bacterial disease that mainly affects the intestinal tract of vertebrate and invertebrate hosts. *Salmonella* bacteria typically live in animal and human intestines and are shed in faeces. Salmonellosis remains a persistent threat to human and animal health and welfare in spite of the advances made in its detection, typing and control (Peek et al. 2004; Refsum et al. 2002). *Salmonella* and salmonellosis cause losses in the livestock and poultry industry because of death, abortion, decreased milk, meat and egg production, cost of testing and control programmes (Uzzau et al. 2000; Veling et al. 2002).

The genus *Salmonella* contains the species *Salmonella enterica, Salmonella bongori* and *Salmonella subterranean,* which was proposed in 2005 (Coburn, Grassl & Finlay 2007). However, according to the World Health Organization, the new species, *Salmonella subterranean*, does not belong to the genus *Salmonella* (Grimont & Weill 2007). *Salmonella enterica* subspecies are further classified into more than 2500 serovars or serotypes and include pathogens of great medical and veterinary importance (EFSA 2008; Mead et al. 1999; Morgan et al. 2004). These serovars differ greatly in their host range and degree of host adaptation. *Salmonella* serotypes are named in accordance with the Kaufmann–White typing system, defined by different combinations of somatic O, surface Vi and flagellar H antigens, which determine the serologically defined names appended to the *Salmonella* species as serovars or serotypes (Popoff 2001). In 2005, *Salmonella enterica* finally gained official approval as the type species of the genus *Salmonella* (Coburn et al. 2007).

The incidence of *Salmonella* infections in animals is closely associated with the age of the animals, husbandry and management – related factors in intensive farming that are conducive to the spread of infection resulting in an increase of clinical disease outbreaks (Baumler et al. 1998; Kidanemariam, Engelbrecht & Picard 2010). Clinically sick animals pose the greatest risk to humans because they are more likely to shed *Salmonella* in higher concentrations than apparently healthy animals.

Salmonellosis is one of the most common food-borne diseases. High-protein foods such as meat, poultry, fish and eggs are most commonly associated with *Salmonella*. However, any food that becomes contaminated and held at temperatures that promote bacterial growth can cause salmonellosis (Altekruse et al. 2006; Kidanemariam et al. 2010; Kimura et al. 2004).

Periodic surveillance reports, such as this one, could provide useful information on the changing patterns of salmonellosis in animals and foods of animal origin. It also assists in the study of the epidemiology, risk factors associated with the disease, and control of the disease. The purpose of this report is to present the retrospective records of the animal isolations and the zoological distribution of the *Salmonella* serotypes isolated and serotyped at the Agricultural Research Council – Onderstepoort Veterinary Research (ARC-OVR) from 2007 to 2014.

## Materials and methods

Specimens submitted to the ARC-OVR bacteriology laboratory comprised bacterial cultures, organs, tissues, swabs, faeces, fluid and tissues obtained after abortion and egg samples. Unprocessed and raw specimens were inoculated into buffered peptone water (pH 7.2) as pre-enrichment and incubated at 37 °C for 18–24 h. One millilitre of the pre-enrichment broth was transferred into 9 ml of Rappaport Vassiliadis (Oxoid^®^) enrichment broth and incubated at 42 °C for 18–24 h. Subcultures from enrichment media were grown on to selective solid media such as xylose-lysine deoxycholate (XLD) agar (Difco^®^) and incubated at 37 °C for 18–24 h. Black colonies with a pink periphery were considered presumptively positive for *Salmonella* and were further confirmed with biochemical tests. Only those Gram-negative isolates that were indole-negative, motile, Simmon’s citrate-negative, urease negative, produced hydrogen sulphide in a triple sugar iron (TSI) slant, were lysine decarboxylase positive, fermented dulcitol but did not ferment lactose and were malonate negative were considered to be *Salmonella enterica.* Additional carbohydrate fermentation tests, such as gas production in Durham tubes and fermentation of sorbitol, arabinose, rhamnose, maltose and trehalose, were included to make provision for those *Salmonella* organisms that do not necessarily fit the above-mentioned criteria. Consideration was also given to *S. enterica* serovar Gallinarum and *S. enterica* serovar Pullorum that are non-motile when isolation and identification were done. There are variable reactions to Simmon’s citrate utilisation and hydrogen production, and special attention was given to these features not to miss salmonellae.

Confirmed *Salmonella* isolates were further serotyped according to the Kauffmann–White classification scheme using a battery of somatic O and flagellar H polyvalent and monovalent antisera.

Data were captured in a dedicated Microsoft Excel^TM^ data sheet for subsequent analysis. Descriptive statistics were employed to obtain values of proportions and percentiles.

## Results

A total of 1229 *Salmonella* isolates were serotyped successfully during the surveillance period 2007–2014. The number of isolates and distribution of the various serotypes in different host species are shown in Table 1. The isolates were classified into 108 serotypes under the 17 groups of the Kauffmann–White classification scheme.

**TABLE 1:** Number of isolates and zoological distribution of the various serotypes 2007–2014.

Serotype	Cattle	Poultry	Leopard	Rhino	Crocodile	Feline	Equine	Sheep and goat	Pig	Total
*S* Aarhus	1	1	0	0	0	0	0	0	0	2
*S* Abaetetuba	0	7	0	0	0	0	0	0	0	7
*S* Aberdeen	3	9	0	0	0	0	0	0	0	12
*S* Achersleben	0	0	0	0	0	0	2	0	0	2
*S* Adeoyo	0	0	0	0	0	0	9	0	0	9
*S* Agona	0	12	0	0	0	0	0	2	0	14
*S* Alachua	0	3	0	0	0	0	0	0	0	3
*S* Anatum	13	13	0	0	0	0	36	0	0	62
*S* Berta	0	7	0	0	0	0	0	0	0	7
*S* Bovismorbificans	1	0	0	0	0	0	0	0	0	1
*S* Blockley	0	2	0	0	0	0	0	0	0	2
*S* Brandenburg	1	3	0	0	0	0	0	0	0	4
*S* Braenderup	0	6	0	0	0	0	0	0	0	6
*S* Brancaster	0	16	0	0	0	0	0	0	0	16
*S* Bredeney	1	3	0	0	0	0	0	0	0	4
*S* Brezany	0	1	0	0	0	0	0	0	0	1
*S* Canada	0	2	0	0	0	0	0	0	0	2
*S* Cardoner	6	0	0	0	0	0	0	0	0	6
*S* Cerro	0	1	0	0	0	0	0	0	0	1
*S* Chailey	0	2	0	0	0	0	0	0	0	2
*S* Chester	2	0	0	0	0	0	0	0	0	2
*S* Chicago	2	0	0	0	0	0	0	0	0	2
*S* Cremieu	1	0	0	0	0	0	0	0	0	1
*S* Claibornei	0	2	0	0	0	0	0	0	0	2
*S* Colorado	0	0	0	0	0	0	4	0	0	4
*S* Corvallis	0	4	0	0	0	0	0	0	0	4
*S* Cubana	1	0	0	0	0	0	0	0	0	1
*S* Derby	0	3	0	0	0	0	0	0	2	5
*S* Dublin	39	0	0	1	0	0	0	2	0	42
*S* Duesseldorf	1	2	0	0	0	0	0	0	0	3
*S* Duisburg	0	2	0	0	0	0	0	0	0	2
*S* Eastbourne	0	1	0	0	0	0	0	0	0	1
*S* Edinburg	0	2	0	0	0	0	0	0	0	2
*S* Eko	0	2	0	0	0	0	0	0	0	2
*S* Enteritidis	1	159	0	3	0	0	0	4	3	170
*S* Farmsen	0	6	0	0	0	0	0	0	0	6
*S* Fulda	0	4	0	0	8	0	0	0	0	12
*S* Give	0	0	0	0	0	0	0	0	1	1
*S* Glasgow	0	0	0	0	0	0	2	0	0	2
*S* Glostrup	0	1	0	0	0	0	0	0	0	1
*S* Gombe	0	1	0	0	0	0	0	0	0	1
*S* Goldcoast	2	0	0	0	0	0	0	0	0	2
*S* Gustavia	0	0	0	0	0	0	1	0	0	1
*S* Hadar	0	12	0	0	0	0	0	0	0	12
*S* Havana	0	5	0	0	0	0	0	0	0	5
*S* Hayindogo	0	4	0	0	0	0	0	0	0	4
*S* Heidelberg	1	133	0	12	0	2	52	0	0	200
*S* Idikan	0	3	0	0	0	0	0	0	0	3
*S* Indiana	0	7	0	0	0	0	0	0	0	7
*S* Infantis	8	41	0	0	0	0	5	3	0	57
*S* Isangi	0	3	0	0	0	0	0	3	0	6
*S* Israel	0	1	0	0	0	0	0	0	0	1
*S* Istanbul	0	1	0	0	0	0	0	0	0	1
*S* Jerusalem	0	2	0	0	0	0	0	0	0	2
*S* Kainji	0	2	0	0	0	0	0	0	0	2
*S* Kalamu	0	1	0	0	0	0	2	0	0	3
*S* Kedougou	2	0	0	0	0	0	0	0	0	2
*S* Kentucky	0	7	0	0	0	0	0	0	0	7
*S* Kiambu	1	4	0	0	0	0	0	0	0	5
*S* Kingston	0	1	0	0	0	0	0	0	0	1
*S* Kotu	0	3	0	0	0	0	0	0	0	3
*S* Kottbus	1	0	0	0	0	0	0	0	0	1
*S* Kouka	0	2	0	0	0	0	0	0	0	2
*S* Lexington	0	1	0	0	0	0	0	0	0	1
*S* Lockleaze	0	2	0	0	0	0	0	0	0	2
*S* London	0	4	0	0	0	0	0	0	0	4
*S* Mandera	0	0	0	0	0	0	1	0	0	1
*S* Manhattan	0	1	0	0	0	0	2	0	0	3
*S* Mbandaka	0	6	0	0	0	0	0	0	0	6
*S* Minnesota	0	25	0	0	0	0	0	0	0	25
*S* Mikawasima	0	0	6	0	0	0	0	0	0	6
*S* Montevideo	0	10	0	0	0	0	0	0	0	10
*S* Muenchen	2	43	0	0	0	0	2	1	0	48
*S* Newport	0	22	0	0	0	0	0	0	0	22
*S* Ohio	0	3	0	0	0	0	0	0	0	3
*S* Orion	1	6	0	0	0	0	0	0	0	7
*S* Oslo	0	1	0	0	0	0	0	0	0	1
*S* Othmarschen	1	2	0	0	0	0	0	0	0	3
*S* Paratyphi	0	5	0	0	0	0	0	0	0	5
*S* Planckendael	0	1	0	0	0	0	0	0	0	1
*S* Poitiers	0	1	0	0	0	0	0	0	0	1
*S* Panama	0	1	0	0	0	0	0	0	0	1
*S* Poona	0	1	0	0	0	0	0	0	0	1
*S* Pretoria	2	3	0	0	0	0	2	0	0	7
*S* Reading	2	2	0	0	0	0	0	0	0	4
*S* Rideau	0	0	0	0	0	0	1	0	0	1
*S* Rissen	0	5	0	0	0	0	0	0	0	5
*S* Ruiru	0	4	0	0	0	0	0	0	0	4
*S* Saintpaul	0	11	0	0	0	0	0	0	0	11
*S* Salford	1	0	0	0	0	0	0	0	0	1
*S* Sambre	0	0	0	0	0	0	1	0	0	1
*S* Sandiego	0	0	0	0	0	0	0	0	1	1
*S* Schoeneberg	1	0	0	0	0	0	2	0	0	3
*S* Schwarzengrund	0	47	0	0	0	0	3	0	0	50
*S* Senftenberg	0	25	0	0	0	0	0	0	0	25
*S* Shubra	0	2	0	0	0	0	0	0	0	2
*S* Stanley	0	1	0	0	0	0	0	0	0	1
*S* Stanleyville	0	13	0	0	0	0	0	0	0	13
*S* Stratford	1	0	0	0	0	0	0	0	0	1
*S* Tallahassee	0	2	0	0	0	0	0	0	0	2
*S* Tennessee	0	7	0	0	0	0	0	0	0	7
*S* Thompson	0	13	0	0	0	0	0	0	0	13
*S* Tsevie	0	2	0	0	0	0	0	0	0	2
*S* Typhimurium	17	66	0	6	0	4	35	9	9	146
*S* Virchow	0	13	0	0	0	0	0	0	0	13
*S* Winneba	0	6	0	0	0	0	0	0	0	6
*S* Wippra	0	4	0	0	0	0	0	0	0	4
*S* Yoruba	0	1	0	0	0	0	0	2	0	3

**Total**	**116**	**871**	**6**	**22**	**8**	**6**	**162**	**26**	**16**	**1233**

Of the total of 1229 isolates with 108 serotypes recorded during the period under review, 871 (70.8%) occurred in poultry and other birds, 162 (13.2%) in horses, 116 (9.4%) in cattle, 26 (2.1%) in sheep and goats, 22 (1.8%) in rhinoceroses, 16 (1.3%) in pigs, 8 (0.6%) in crocodiles, 6 (0.5%) in cats and 6 (0.5%) in leopards. Despite the large number of serotypes involved, the majority of isolates were limited to only a few serotypes. For example, from the total of 108 serotypes detected, only 8 serotypes contributed 63.0% (775 isolates), while 100 serotypes collectively accounted for 37.0% (454 isolates). The eight most common serotypes were *S*. Anatum (5.0%), *S*. Dublin (3.4%), *S*. Enteritidis (13.8%), *S*. Heidelberg (16.2%), *S*. Infantis (4.6%), *S*. Muenchen (3.9%), *S*. Schwarzengrund (4.0%) and *S*. Typhimurium (11.8%) (Table 1). The most frequently isolated serotype was *S*. Heidelberg, accounting for 16.2% of all isolates. Twenty-seven serotypes were isolated only once during the survey period. *S*. Typhimurium had the widest zoological distribution, followed by *S.* Enteritidis and *S*. Heidelberg.

In domestic fowl and other birds, the most common serotypes detected were *S*. Enteritidis (18.2%), *S*. Heidelberg (15.2%), *S*. Typhimurium (7.5%) and *S*. Schwarzengrund (5.4%); these four serotypes in total contributed 46.5% of the isolates, while the remaining 84 serotypes accounted for 53.5% of the total isolates (Tables 1 and 2).

**TABLE 2:** Common *Salmonella* serotypes in poultry in South Africa.

Isolate	2007	2008	2009	2010	2011	2012	2013	2014	Total
***S* Enteritidis**									
Count	26	14	10	62	2	12	31	2	159
%	16.88	14.00	10.30	42.42	6.89	7.69	19.25	8.33	18.25
***S* Heidelberg**									
Count	20	0	11	5	0	63	34	0	133
%	12.98	0.00	11.34	3.42	0.00	40.38	21.11	0.00	15.26
***S* Infantis**									
Count	1	6	5	4	8	3	8	6	41
%	0.64	6.00	5.15	2.73	27.58	1.92	4.96	25.00	4.70
***S* Minnesota**									
Count	2	6	1	3	0	4	9	0	25
%	1.29	6.00	1.03	2.05	0.00	2.56	5.59	0.00	2.87
***S* Muenchen**									
Count	2	15	0	2	0	17	7	0	43
%	1.29	15.00	0.00	1.36	0.00	10.89	4.34	0.00	4.93
***S* Newport**									
Count	0	5	3	0	3	5	3	3	22
%	0.00	5.00	3.09	0.00	10.34	3.20	1.86	12.50	2.52
***S* Schwarzengrund**									
Count	0	5	2	8	2	5	18	7	47
%	0.00	5.00	2.06	5.47	6.89	3.20	11.18	29.16	5.39
***S* Senftenberg**									
Count	3	1	15		5	0	1	0	25
%	1.94	1.00	15.46	0.00	17.24	0.00	0.62	0.00	2.87
***S* Typhimurium**									
Count	22	8	20	18	1	9	7	0	66
%	14.28	8.00	20.61	12.32	3.44	5.76	4.34	0.00	7.57
**Others**									
Count	78	48	34	44	16	41	43	6	310
%	50.64	48.00	35.05	30.13	55.17	26.28	26.70	25.00	35.59

**Total**									
**Count**	**154**	**100**	**97**	**146**	**29**	**156**	**161**	**24**	**871**
**%**	**100.00**	**100.00**	**100.00**	**100.00**	**100.00**	**100.00**	**100.00**	**100.00**	**100.00**

In cattle, the most common serotypes detected were *S*. Dublin (33.6%), *S*. Typhimurium (14.6%), *S*. Anatum (11.2%) and *S*. Infantis (6.9%) and accounted for 66.4% of the total isolates, while the remaining 25 serotypes accounted for 33.6% of the total isolates (Table 3).

**TABLE 3:** Common *Salmonella* serotypes in cattle in South Africa.

Isolate	2007	2008	2009	2010	2011	2012	2013	2014	Total
***S* Anatum**									
Count	1	5	1	0	0	5	1	0	13
%	5.00	27.77	6.66	0.00	0.00	33.33	4.00	0.00	11.20
***S* Dublin**									
Count	8	2	6	9	3	2	6	3	39
%	40.00	11.11	40.00	60.00	75.00	13.33	24.00	75.00	33.60
***S* Infantis**									
Count	1	2	0	0	0	2	3	0	8
%	5.00	11.11	0.00	0.00	0.00	13.33	12.00	0.00	6.89
***S* Typhimurium**									
Count	7	4	1	1	0	3	1	0	17
%	35.00	22.22	6.66	6.66	0.00	20.00	4.00	0.00	14.65
**Others**									
Count	3	5	7	5	1	3	14	1	39
%	15.00	27.77	46.66	33.30	25.00	20.00	56.00	25.00	33.62

**Total**									
**Count**	**20**	**18**	**15**	**15**	**4**	**15**	**25**	**4**	**116**
**%**	**100.00**	**100.00**	**100.00**	**100.00**	**100.00**	**100.00**	**100.00**	**100.00**	**100.00**

In horses, 162 isolations involving 18 serotypes were recorded in the period under review. The three major serotypes were *S*. Heidelberg (32.1%), *S*. Anatum (22.2%) and *S*. Typhimurium (21.6%). The remaining 15 serotypes collectively accounted for 24.8% of the total isolations (Tables 1 and 4).

**TABLE 4:** Common *Salmonella* serotypes in horses in South Africa.†

Isolate	2008	2009	2011	2012	2013	2014	Total
***S* Achersleben**							
Count	0	0	1	0	0	1	2
%	0.00	0.00	1.72	0.00	0.00	2.12	1.23
***S* Adeoyo**							
Count	0	0	0	0	9	0	9
%	0.00	0.00	0.00	0.00	20.45	0.00	5.56
***S* Anatum**							
Count	4	0	8	4	12	8	36
%	100.00	0.00	13.79	100.00	27.27	17.02	22.22
***S* Colorado**							
Count	0	0	2	0	0	2	4
%	0.00	0.00	3.45	0.00	0.00	4.25	2.47
***S* Glasgow**							
Count	0	0	0	0	2	0	2
%	0.00	0.00	0.00	0.00	4.54	0.00	1.23
***S* Gustaria**							
Count	0	0	0	0	1	0	1
%	0.00	0.00	0.00	0.00	2.27	0.00	0.62
***S* Heidelberg**							
Count	0	0	32	0	0	20	52
%	0.00	0.00	55.17	0.00	0.00	42.55	32.1
***S* Infantis**							
Count	0	0	0	0	5	0	5
%	0.00	0.00	0.00	0.00	11.36	0.00	3.08
***S* Kalamu**							
Count	0	0	1	0	0	2	2
%	0.00	0.00	1.72	0.00	0.00	4.25	1.23
***S* Mandera**							
Count	0	0	0	0	1	0	1
%	0.00	0.00	0.00	0.00	2.27	0.00	0.61
***S* Mbandaka**							
Count	0	0	1	0	0	1	2
%	0.00	0.00	1.72	0.00	0.00	2.12	1.23
***S* Muenchen**							
Count	0	0	1	0	0	1	2
%	0.00	0.00	1.72	0.00	0.00	2.12	1.23
***S* Pretoria**							
Count	0	0	0	0	2	0	2
%	0.00	0.00	0.00	0.00	4.54	0.00	1.23
***S* Rideau**							
Count	0	0	0	0	1	0	1
%	0.00	0.00	0.00	0.00	2.27	0.00	0.61
***S* Sambre**							
Count	0	0	0	0	1	0	1
%	0.00	0.00	0.00	0.00	2.27	0.00	0.61
***S* Schoeneberg**							
Count	0	0	1	0	0	1	2
%	0.00	0.00	1.72	0.00	0.00	2.12	1.23
***S* Schwarzengrund**							
Count	0	1	0	0	2	0	3
%	0.00	16.70	0.00	0.00	4.54	0.00	1.85
***S* Typhimurium**							
Count	0	5	11	0	8	11	35
%	0.00	83.33	18.96	0.00	18.18	23.40	21.60

**Total**							
**Count**	**4**	**6**	**58**	**4**	**44**	**47**	**162**
**%**	**100.00**	**100.00**	**100.00**	**100.00**	**100.00**	**100.00**	**100.00**

† , No samples were presented for testing between 2007 and 2010.

Eight serotypes were identified from 26 isolations that occurred in sheep and goats. *Salmonella* Typhimurium (34.6%), *S*. Enteritidis (15.4%), *S*. Infantis (11.5%) and *S*. Isangi (11.5%) were the major serotypes and collectively accounted for 73.1% of the isolations (Table 5).

**TABLE 5:** Common *Salmonella* serotypes in sheep and goats in South Africa.

Isolate	2010	2012	2013	Total
***S* Agona**				
Count	0	1	1	2
%	0.00	14.28	7.69	7.69
***S* Dublin**				
Count	0	0	2	2
%	0.00	0.00	15.38	7.69
***S* Enteritidis**				
Count	1	1	2	4
%	16.66	14.28	15.38	15.38
***S* Infantis**				
Count	0	0	3	3
%	0.00	0.00	23.07	11.53
***S* Isangi**				
Count	1	2	0	3
%	16.66	28.57	0.00	11.53
***S* Muenchen**				
Count	0	1	0	1
%	0.00	14.28	0.00	3.84
***S* Typhimurium**				
Count	3	1	5	9
%	50.00	14.28	38.46	34.61
***S* Yoruba**				
Count	1	1	0	2
%	16.66	14.28	0.00	7.69

**Total**				
**Count**	**6**	**7**	**13**	**26**
**%**	**100.00**	**100.00**	**100.00**	**100.00**

Sixteen isolations involving five serotypes were recorded in pigs. The majority of isolates were *S*. Typhimurium, that accounted for 56.3% of the cases, followed by *S*. Enteritidis (18.7%) and *S.* Derby (12.5%) (Table 6). In captive and wild animals, 42 isolates involving 6 serotypes were recorded (Table 7). *Salmonella* Heidelberg (33.33%) and *S.* Typhimurium (23.8%) were the two major serotypes isolated during the survey period. *Salmonella* Fulda (19.0%) and *S*. Mikawasima (14.3%) were isolated exclusively from crocodiles and leopards, respectively (Table 7). *Salmonella* Dublin (2.4%) and *S*. Enteritidis (7.1%) were detected from rhinoceroses.

**TABLE 6:** Common *Salmonella* serotypes in pigs in South Africa.

Isolate	2008	2009	2010	2011	2012	2013	2014	Total
***S* Derby**								
Count	1	0	0	0	1	0	0	2
%	20.00	0.00	0.00	0.00	20.00	0.00	0.00	12.50
***S* Enteritidis**								
Count	1	0	0	0	1	1	0	3
%	20.00	0.00	0.00	0.00	20.00	25.00	0.00	18.75
***S* Give**								
Count	0	0	0	0	0	1	0	1
%	0.00	0.00	0.00	0.00	0.00	25.00	0.00	6.25
***S* Sandiego**								
Count	0	0	1	0	0	0	0	1
%	0.00	0.00	100.00	0.00	0.00	0.00	0.00	6.25
***S* Typhimurium**								
Count	3	0	0	0	3	2	1	9
%	60.00	0.00	0.00	0.00	60.00	50.00	100.00	56.25

**Total**								
**Count**	**5**	**0**	**1**	**0**	**5**	**4**	**1**	**16**
**%**	**100.00**	**0.00**	**100.00**	**0.00**	**100.00**	**100.00**	**100.00**	**100.00**

**TABLE 7:** Common *Salmonella* serotypes in wild and captive animals in South Africa.†

Isolate	2011	2012	2013	2014	Total
***S* Dublin**					
Count	0	0	0	1	1
%	0.00	0.00	0.00	4.76	2.38
***S* Enteritidis**					
Count	1	0	1	1	3
%	5.00	0.00	100.00	4.76	7.14
***S* Fulda**					
Count	4	0	0	4	8
%	20.00	0.00	0.00	19.04	19.04
***S* Mikawasima**					
Count	3	0	0	3	6
%	15.00	0.00	0.00	14.28	14.28
***S* Heidelberg**					
Count	7	0	0	7	14
%	35.00	0.00	0.00	33.33	33.33
***S* Typhimurium**					
Count	5	0	0	5	10
%	25.00	0.00	0.00	23.81	23.81

**Total**					
**Count**	**20**	**0**	**1**	**21**	**42**
**%**	**100.00**	**0.00**	**100.00**	**100.00**	**100.00**

† , No samples were presented for testing between 2007 and 2010.

## Discussion

Laboratory diagnosis continues to provide important epidemiological information that contributes significantly to continuous disease surveillance programmes in the country, if properly validated tests are used. The data set presented in this report is a follow-up of similar laboratory surveillance information carried out from 1996 through 2006 as described elsewhere (Kidanemariam et al. 2010).

There has been a steady increase over the years in the number of *Salmonella* serotypes isolated and in the number of animal species involved. *Salmonella* serotypes clearly seem to differ in their distributions between different animal populations (Table 1). For instance, approximately 95.3% of all detected *Salmonella* serotypes are predominantly associated with domestic species, namely poultry, cattle, horses and sheep and goats, whereas rhinoceroses, leopards, crocodiles and pigs contributed only 4.7%. However, care must be taken when considering these data because of the possibility of a skewed focus from targeted sampling of mostly food animals as compared to wild and game animals. In spite of this, the data probably give a reasonable assessment of the incidence of salmonellosis and the *Salmonella* serotypes involved.

Over 2500 *Salmonella* serovars are recognised worldwide (Coburn et al. 2007; EFSA 2008), and the number continues to rise. However, despite the existence of formidable number of different serotypes, only a few are commonly associated with clinical disease in humans and animals (Warnick et al. 2001). It is also noted in the current study that despite the large number of serotypes involved, the majority of isolates were mainly represented by a small number of serotypes. For instance, from the total of 108 serotypes detected among 1229 isolates, only 8 serotypes contributed 63.0 % (775 isolates), while 100 serotypes collectively accounted for 37.0% (454 isolates).

*Salmonella* serotypes can be divided into host-specific, host-adapted and generalist serotypes, with important implications for the epidemiology and risk factors of the diseases in the associated host species (Baumler et al. 1998; Uzzau et al. 2000).

Host-specific serotypes, such as *S*. Paratyphi A in humans and *S*. Gallinarum biovars Gallinarum and Pullorum in chickens, only caused disease in one host species (Pascopella et al. 1995; Uzzau et al. 2000). These host-specific serotypes were not isolated during the survey period. *Salmonella* Paratyphi A is exclusively a human pathogen, and samples are submitted to public health facilities. Nonetheless, *S*. Typhi was confirmed through blood and stool cultures from cases of a typhoid fever outbreak in South Africa (Anon 2016). The absence of *S*. Gallinarum and *S*. Pullorum in the data set of the current survey could be because of the regular targeted monitoring programme that assisted poultry farms to control and prevent *Salmonella* infections in general and infections as a result of *S*. Gallinarum and *S*. Pullorum in particular (A.K. Gelaw, unpublished results). In addition, small incidences are less likely to be reported and to be passed unnoticed. Furthermore, the lack of either serovar in the current survey may also be attributed to the selection criteria in the methodology that often targets motile salmonellae.

**FIGURE 1:**
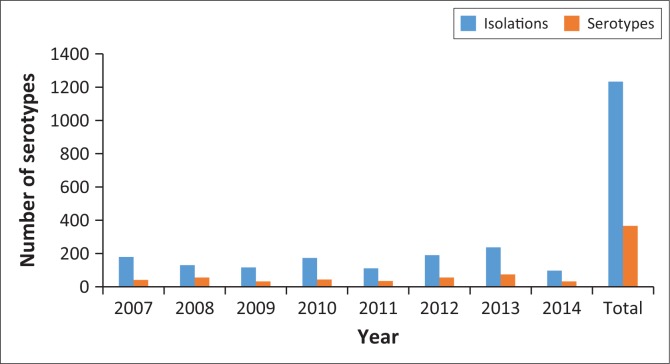
Annual *Salmonella* isolations and number of serotypes from animal origin.

Host-adapted serotypes are predominantly associated with a single host species, but reports suggest that these serotypes can cause disease in other species as well (Pascopella et al. 1995; Uzzau et al. 2000). For example, *S.* Dublin, *S*. Enteritidis and *S*. Choleraesuis were previously regarded as host-adapted serotypes for cattle, chicken and pigs, respectively (Anderson et al. 1997; Uzzau et al. 2000). However, it became apparent that these serotypes commonly cause disease in a broad range of hosts and may be considered as generalist serotypes. This is supported by the results of the current survey where *S.* Dublin was isolated from cattle, goats and rhinoceroses, and *S*. Enteritidis was isolated from poultry, pigs, sheep and rhinoceroses, demonstrating the ability of these serotypes to adapt and infect multiple hosts.

Generalist serotypes such as *Salmonella* Typhimurium, on the other hand, are associated with a wide range of hosts. The current data supported this assertion, as *S*. Typhimurium was detected across a wide range of animal species that include poultry, cattle, horses, sheep, goats and pigs (Table 1). This serotype was also found in wild and captive animals such as a leopard, rhinoceros and crocodile. Similar studies have demonstrated that *S*. Typhimurium is the most common serovar isolated in livestock (Morgan et al. 2004; Rabsch et al. 2002) and human non-typhoidal infections, especially in immune-compromised patients (Calvert, Stewart & Reilly 1998; Gordon 2008; Keddy et al. 2017; Voetsch et al. 2004).

*Salmonella* Heidelberg is one of the generalist serotypes and was by far the most common serotype, representing 16.2% of the total isolations, with the majority originating from horses, in which species it accounted for 32.1% of the total cases. This result could partially be attributed to the increased epidemiological monitoring to ascertain the causes of clinical episodes of enteric infections in the hospitalised horses at the Veterinary Academic Hospital of the University of Pretoria (J. Gouws, pers. com., June 2015). Faecal material was submitted for testing, and *S*. Heidelberg was isolated from horses admitted to the hospital. Because of the potential risk of spread of *Salmonella* from an infected horse, the equine hospital has introduced biosecurity measures to help prevent disease transmission to personnel, the environment and other patients. Similar reports showed that hospitalised horses were more likely to shed *Salmonella* than horses housed in their own barns, possibly because of stress or illness (Amavisit et al. 2001). A study at a large veterinary hospital in the United States demonstrated that 13% of horses admitted for colic were shedding *Salmonella* species (Kim et al. 2001).

*Salmonella* Typhimurium exhibited the widest zoological distribution (Table 1). This finding is not unexpected because *S*. Typhimurium has been thought of as the most ubiquitous and broadest host-range serotype, as it is frequently associated with diseases in numerous host species, including humans, livestock, domestic fowl, rodents and birds (Bahnson et al. 2006; Padungtod, Kadohira & Hill 2008). It was, however, demonstrated in the current survey that the incidence of *S*. Typhimurium was relatively higher in poultry and equines with incidence rates of 45.2% and 23.9%, respectively.

Although *S*. Enteritidis was isolated from various host species, it was by far the most common serotype encountered in poultry, accounting for 93.5% of the incidents. The remaining 6.5% were shared among cattle (0.6%), rhinoceros (1.7%), sheep (2.3%) and pigs (1.7%). This serotype is among the most common pathogens of chicken that could also adversely affect the health of human beings and other animal species following exposures (Kidanemariam et al. 2010). Vertical transmission with internal transovarian contamination of egg-yolk with *S*. Enteritidis has been confirmed, making uncooked eggs no longer safe to eat (Altekruse et al. 1993). Furthermore, *S*. Enteritidis was confirmed as causes of human outbreaks of non-typhoidal salmonellosis associated with the consumption of foods of animal origin in South Africa (Muvhali et al. 2017). *Salmonella* Enteritidis is often presented separately from other serotypes of *Salmonella* because this bacterium is specifically cited in zoonosis control legislation. The South African government has included *S*. Enteritidis in the list of controlled diseases (Anon 1984; Kidanemariam et al. 2010).

*Salmonella* Dublin and *Salmonella* Typhimurium are the two predominant serotypes detected in cattle in the current survey, accounting for 48.3% of the total recorded isolations. The relative incidence of *S*. Dublin (33.6%) is, however, higher than that of *S*. Typhimurium (14.6%) for the survey period. Similar studies have shown that *S*. Dublin may potentially be the most frequently isolated serotype in cattle, more than the broad host-range serotypes, such as *S*. Typhimurium (Brackelsberg, Nolan & Brown 1997; Kidanemariam et al. 2010). It should be noted that the disease epidemiology of *S*. Dublin and *S*. Typhimurium varies considerably. Most importantly, cattle infected with *S*. Dublin often become asymptomatic carriers and continue to excrete large numbers of organisms in their faeces for many years and often for life (Giles, Hopper & Wray 1989; Rice, Besser & Hancock 1997; Vanselow et al. 2007). Effective control of infection should thus include removal of chronically infected cattle and implementation of stringent biosecurity measures. On the other hand, hosts infected with *S*. Typhimurium will only shed the organism for a few weeks to a month after clinical recovery (Huston, Wittum & Love 2002). However, it should be noted that *S*. Typhimurium tends to persist in the environment for longer periods (Rabsch et al. 2002). One study estimated the median duration of shedding in dairy cattle to equal 50 days, with a maximum duration of 391 days (Cummings et al. 2009). This can be mitigated by implementing proper biosecurity measures at all times.

Clinically affected herds and certain management systems may pose an increased risk to public health. Little is known about the *Salmonella* risk posed to humans by indirect animal contact, especially through environmental contamination. Considering the public health significance of this group of bacteria, further studies, especially on spatial clustering of human cases around livestock premises, are needed to assess the indirect risks posed by livestock operations in South Africa.
